# *har-1/CHCHD10* mutations induce neurodegeneration and mitochondrial fragmentation in *Caenorhabditis elegans*

**DOI:** 10.17912/micropub.biology.001597

**Published:** 2025-05-15

**Authors:** Audrey Labarre, Ericka Guitard, Gilles Tossing, J Alex Parker

**Affiliations:** 1 Centre de recherche du centre hospitalier de l'Université de Montréal (CRCHUM); 2 Neurosciences, Université de Montréal, Montréal, Quebec, Canada

## Abstract

*CHCHD10* encodes a mitochondrial protein that plays a role in cristae morphology and oxidative phosphorylation, with mutations associated with neurodegenerative diseases, including the spectrum of amyotrophic lateral sclerosis and frontotemporal dementia (ALS-FTD). The *Caenorhabditis elegans* ortholog of *CHCHD10* is *har-1* , which can be used to model CHCHD10-related neurodegenerative diseases. We focused on two *har-1* mutant strains: one featuring a 260 bp deletion ( *gk3124* ) and the other with a G73E point mutation ( *ad2155* ). Both *har-1* mutants displayed progressive paralysis, degeneration of GABAergic motor neurons, and mitochondrial fragmentation. These strains may be valuable tools for investigating pathogenic mechanisms and therapeutic strategies for neurodegenerative diseases.

**Figure 1. Phenotypic characterization of har-1 disruption:**
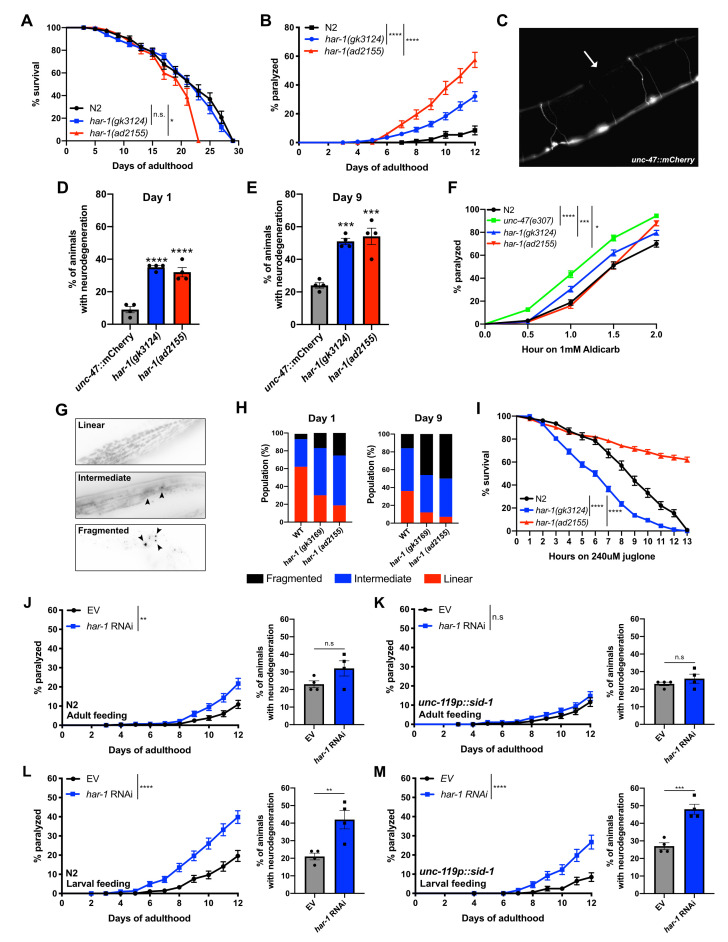
( **A** ) *har-1 ( gk3124 )* does not affect lifespan, while the *har-1 ( ad2155 )* mutation shortens lifespan compared to N2 (Log-rank (Mantel-Cox) test). ( **B** ) *har-1 ( gk3124 )* and *har-1 ( ad2155 )* mutants exhibited motility defects leading to age-dependent paralysis when compared to N2 worms (Log-rank (Mantel-Cox) test). ( **C** ) A representative image shows GABAergic neurodegeneration observed in *har-1 ( ad2155 )* mutants, with the white arrow indicating a neuronal gap indicative of neurodegeneration. *har-1 ( gk3124 )* and *har-1 ( ad2155 )* worms display GABAergic motor neuron degeneration at ( **D** ) day 1 and ( **E** ) day 9 (One-way ANOVA followed by Dunnett's multiple comparisons test). ( **F** ) *har-1 ( gk3124 ) and har-1 ( ad2155 )* worms are more sensitive to aldicarb-induced paralysis than N2 worms, although not to the same extent as *unc-47 ( e307 )* animals (Log-rank (Mantel-Cox) test). ( **G** ) Representative images of transgenic animals expressing *TOM20::mRFP* in body-wall muscle cells show mitochondrial organization quantified as either “Linear,” “Intermediate,” or “Fragmented.” *har-1* mutants exhibited higher levels of mitochondrial impairment compared to N2 animals at ( **H** ) day 1, Linear: N2 vs. *har-1 ( gk3124 )* **** *P* <0.0001, N2 vs. *har-1 ( ad2155 )* **** *P* <0.0001; Intermediate: N2 vs. *har-1 ( gk3124 )* *** *P* <0.001, N2 vs. *har-1 ( ad2155 )* *** *P* <0.001, Fragmented: N2 vs. *har-1 ( gk3124 )* n.s., N2 vs. *har-1 ( ad2155 )* ** *P* <0.01) and day 9 (Linear: N2 vs. *har-1 ( gk3124 )* **** *P* <0.0001, N2 vs. *har-1 ( ad2155 )* **** *P* <0.0001; Intermediate: N2 vs. *har-1 ( gk3124 )* n.s., N2 vs. *har-1 ( ad2155 )* n.s., Fragmented: N2 vs. *har-1 ( gk3124 )* **** *P* <0.0001, N2 vs. *har-1 ( ad2155 )* **** *P* <0.0001) (Two-way ANOVA followed by Dunnett's multiple comparisons test) ( **I** ) *har-1 ( gk3124 )* worms were hypersensitive to oxidative stress, while *har-1 ( ad2155 )* mutants displayed resistance to acute juglone exposure (Log-rank (Mantel-Cox) test). ( **J** ) N2 animals were slightly paralyzed when fed RNAi against *har-1* starting at day 1 of adulthood but did not exhibit GABAergic neurodegeneration (Log-rank (Mantel-Cox) test and One-way ANOVA followed by Dunnett's multiple comparisons test). ( **K** ) Neuronal *har-1* RNAi knockdown in worms beginning at day 1 of adulthood did not result in motility issues or neurodegeneration (Log-rank (Mantel-Cox) test and One-way ANOVA followed by Dunnett's multiple comparisons test). ( **L** ) N2 worms and ( **M** ) *unc-119 p:: sid-1* transgenics displayed age-dependent paralysis and GABAergic neurodegeneration when given RNAi against *har-1* starting at the L1 stage. *n.s.* : non-significant, *: *P* < 0.05, **: *P* < 0.01, ***: *P* < 0.001, ****: *P* < 0.0001 (Log-rank (Mantel-Cox) test and One-way ANOVA followed by Dunnett's multiple comparisons test).

## Description

*CHCHD10* is one of several genes associated with the spectrum of ALS-FTD neurodegenerative diseases (Abramzon et al., 2020). There are no cures for ALS-FTD, and current treatments have modest effects, underscoring the need to develop models for therapeutic discovery. *CHCHD10* encodes a coiled-coil-helix-coiled-coil-helix protein found in the intermembrane space of mitochondria, playing roles in maintaining mitochondrial cristae morphology, oxidative phosphorylation, and synaptic integrity (Bannwarth et al., 2014; Woo et al., 2017).

The *C. elegans* HAR-1 amino acid sequence shows 41% identity, 12% conserved similarity, and 18% semi-conserved similarity with human CHCHD10 (Woo et al., 2017). *C. elegans* has been used to model aspects of ALS-FTD, so we investigated *har-1* mutations' effects on survival, motility, neurodegeneration, and mitochondrial morphology. Mutants *har-1 ( gk3124 )* and *har-1 ( ad2155 )* appeared normal, but *har-1 ( ad2155 )* had a shorter lifespan than control N2 worms ( **Fig. 1A** ). Both mutants showed age-dependent paralysis starting around day 6, with *ad2155* displaying a stronger phenotype than *gk3124* ( **Fig. 1B** ). We evaluated paralysis links to GABAergic motor neuron degeneration and found significant neuron loss in both mutants, especially in the dorsal cord ( **Fig. 1C** ). Neuron degeneration progressed, with day 1 animals ( **Fig. 1D** ) losing fewer neurons than day 9 animals ( **Fig. 1E** ).

Previous work shows *C. elegans* ALS models often exhibit aldicarb hypersensitivity, highlighting synaptic function changes (Therrien et al., 2013; Vaccaro et al., 2012). To assess if *har-1* mutants displayed similar traits, worms were treated with 1 mM aldicarb, an acetylcholinesterase inhibitor for investigating dysfunctional neuromuscular transmission (Mahoney et al., 2006). Worms like *unc-47 ( e307 )* show hypersensitivity to aldicarb-induced paralysis (Vashlishan et al., 2008). After treatment, *har-1 ( gk3124 )* and *har-1 ( ad2155 )* mutants paralyzed quicker or more frequently than N2 ( **Fig. 1F** ). However, their paralysis rates were not as high as the positive control *unc-47 ( e307 )* . These findings suggest that *har‑1* mutations disrupt neuromuscular transmission—a defect common in some ALS‑FTD cases (Ng et al., 2015). Since aldicarb acts primarily by inhibiting acetylcholinesterase, the altered sensitivity could reflect impaired acetylcholine release, altered postsynaptic receptor function, or modulation by other neurotransmitter systems. Further experiments are required to elucidate the precise underlying mechanism.

Given CHCHD10's mitochondrial functions, we examined *har-1* mutants for mitochondrial morphology (Bannwarth et al., 2014). Using a TOM20::mRFP reporter, we assessed mitochondrial fragmentation in body-wall muscle cells. In young wild-type worms, the mitochondrial network mostly appears as linear fragments, with some showing disorganized shapes, indicating disrupted structure. We categorized organization as “Linear,” “Intermediate,” or “Fragmented” ( **Fig. 1G** ) (Sarasija and Norman, 2018; Sarasija and Norman, 2015). Both *har-1* mutants showed fewer “Linear” structures and more “Intermediate” and “Fragmented” types at day 1 of adulthood compared to N2 worms ( **Fig. 1H** ). Aging worsened the loss of organization, with adult day 9 worms exhibiting more “Fragmented” phenotypes ( **Fig. 1H** ).

Next, we investigated the role of *har-1* and oxidative stress. The natural compound juglone induces oxidative stress and mortality in *C. elegans (* Tauffenberger and Parker, 2014 *)* . We observed that *har-1 ( gk3124 )* mutants were hypersensitive to acute exposure to juglone ( **Fig. 1I** ). Surprisingly, *har-1 ( ad2155 )* worms demonstrated high resistance to juglone-induced mortality stress ( **Fig. 1I** ). These data show that *har-1* mutations promote motility and mitochondrial deficits, but the mutations are not equivalent in their response to oxidative stress. These results imply that mutations in *har-1* lead to mitochondrial deficits, which correlate with neurodegeneration and motor phenotype.

Whether *CHCHD10* mutations associated with ALS-FTD follow a loss-of-function or a gain-of-function mechanism remains unclear. We tested whether RNA interference (RNAi) depletion of *har-1* could induce ALS-FTD phenotypes. N2 worms treated with *har-1* RNAi from day 1 of adulthood exhibit age-dependent paralysis starting around day 8, without significant GABAergic neurodegeneration by day 9 ( **Fig. 1J** ). In *C. elegans* , neuronal cells resist RNAi and require genetic modification for sensitivity. We utilized a neuronal-sensitive RNAi strain, *unc-119 :: sid-1 (* Calixto et al., 2010 *)* . RNAi that targeted *har-1* in *unc-119 :: sid-1* worms on day 1 did not result in paralysis or degeneration ( **Fig. 1K** ). To evaluate the developmental effects of *har-1* depletion, we administered *har-1* RNAi to N2 and *unc-119 :: sid-1* worms starting from the L1 larval stage. Both non-neuronal and neuronal larval knockdown resulted in age-dependent paralysis with significant GABAergic neurodegeneration by day 9 ( **Fig. 1L- M** ). N2 worms displayed more severe paralysis, while motor neuron degeneration post- *har-1* depletion was comparable in both strains. These findings suggest that larval *har-1* depletion at a critical stage, particularly in neurons, impacts ALS-FTD-related phenotypes later in life.

In conclusion, *har-1* mutants and RNAi methods may serve as valuable tools for studying *CHCHD10* -related pathogenic mechanisms, drug screening, and exploring potential developmental factors in age-dependent neurodegeneration.

## Methods

***C* . *elegans* maintenance and strains**

Standard *C. elegans* culturing and handling methods were employed (Stiernagle, 2006). Worms were grown on N.G.M. agar plates streaked with *E* . *coli* OP50 . All assays were conducted at 20 °C. Strains were sourced from the *C. elegans* Genetics Center (University of Minnesota, Minneapolis) and include the N2 Bristol strain, VC3169 ( *har-1 ( gk3124 ))* , DA2155 ( *har-1 ( ad2155 ))* , CB307 ( *unc-47 ( e307 ))* , PS6192 ( *syIs243 [myo-3p::TOM20::mRFP + unc-119 (+) + pBS Sk+]),* and TU3311 ( *uls60 [ unc-119 p::YFP + unc-119 p:: sid-1 ]).* The IZ629 ( *ufsl34 [P unc-47 p::mCherry])* strain was kindly provided by Dr. Michael M. Francis (University of Massachusetts, Worcester, MA). Before use, mutant strains underwent four rounds of outcrossing to N2 . These crosses led to the development of additional *C. elegans* strains. PCR or sequencing was utilized to confirm the homozygosity of each genotype. For each experiment, synchronized populations were maintained on fresh NGM plates by manually transferring adults every other day to prevent progeny from confounding the aging cohorts. No FUDR was used. All data acquisition and scoring were performed with the experimenter blinded to worm genotype.

**Solid media paralysis assay**

From day 1 to day 12 of adulthood, 40 age-synchronized L4 worms were transferred to NGM plates and examined daily for signs of paralysis. If a worm did not move its head after being tapped on the nose and showed no pharyngeal pumping, it was considered dead. Animals that did not respond after being prodded with a worm pick were deemed paralyzed. Each experiment was conducted three times in duplicate at 20° C.

**Lifespan assay**

Worms were grown on NGM. From day one of adulthood until death, approximately 40 age-synchronized L4 worms were examined every two days. Experiments were conducted in triplicate at 20 °C. If worms did not move or respond to tactile stimuli, they were recorded as dead. Excluded from the study were dead worms with internally developed eggs, gonads protruding from their bodies, or worms that crawled off the plate.

**Aldicarb test**

Worms were grown on NGM plates and transferred to plates containing 1 mM aldicarb on day 1 of adulthood. Animals were scored every 30 minutes for two hours and counted as paralyzed if they failed to move after being prodded on the nose. Thirty animals per plate were tested in triplicates at 20° C.

**RNAi experiments**

Strains treated with RNA interference (RNAi) were fed *E. coli* ( HT115 ) containing an empty vector (L4440 (pPD129.36)) or *har-1* ( C16C10.11 ), sourced from the *C. elegans* RNAi Ahringer library (Source BioScience). RNAi experiments were conducted at 20 °C, and the worms were grown on NGM enriched with 1 mM isopropyl-ß-D-thiogalactopyranoside (IPTG).

**Neurodegeneration assay**

Worms were selected on days 1 and 9 of adulthood for *in vivo* motor neuron imaging to assess neural processes for gaps or breakage. The animals were placed onto plates with 2% agarose pads after being immobilized in 5 mM levamisole dissolved in M9. Visualization was conducted using a Zeiss Axio Imager M2 microscope, equipped with a 20X objective and a 1.5 Optovar, along with AxioVs40 4.8.2.0. software. Across four distinct studies, at least 100 worms were analyzed for each condition.

**Oxidative stress assay**

Worms were cultivated on standard NGM plates until the first day of adulthood. Thirty day 1 adults were then transferred to NGM plates containing 240 µM juglone. Tests were conducted at 20° C. At least thirty animals per plate in triplicates were tested at 20 °C. Worms were counted every hour for up to 13 hours, and they were considered dead if they did not respond to prodding.

**Mitochondrial morphology analysis**

The body-wall mitochondrial structure was analyzed using the PS6192 [ *Pmyo-3* ::TOM20::mRFP + *unc-119* (+) + pBS SK+] strain, which targets a red fluorescent protein (mRFP) to the outer mitochondrial membrane. This strain was crossed into both *har-1* genotypes to assess mitochondrial health. Age-synchronized day 1 and day 9 adults were immobilized with 5 mM levamisole on a 2% agarose pad. Visualization was performed using a Zeiss Axio Imager M2 microscope, a 40X objective, and a 1.5 Optovar. The software utilized was AxioVs40 4.8.2.0. Mitochondria were categorized as linear, intermediate, or fragmented, as previously described (Lu et al., 2011). At least one hundred worms were analyzed per condition across four distinct experiments.

**Statistics**

Survival curves were created and compared for paralysis, lifespan, and stress resistance assays using the Log-rank (Mantel-Cox) test, with 60–100 worms examined per genotype at least three times. For the neurodegeneration assay, a one-way ANOVA followed by Dunnett's multiple comparisons test was conducted. A two-way ANOVA followed by Dunnett's multiple comparisons test was conducted for mitochondrial morphology. Quantitative data were expressed as mean ± S.E.M., except for mitochondrial morphology data, where error bars were removed from the graph for clarity. All statistical evaluations were performed using GraphPad Prism v8 software.

## Reagents

**Table:** 

**Strain**	**Genotype**	**Available from**
N2	Wild type	CGC
VC3169	*har-1 ( gk3124 ) *	CGC
DA2155	*har-1 ( ad2155 ) *	CGC
CB307	*unc-47 ( e307 ) *	CGC
PS6192	*syIs243* * [myo-3p::TOM20::mRFP + unc-119 (+) + pBS Sk+] *	CGC
TU3311	* uls60 [ unc-119 p::YFP + unc-119 p:: sid-1 ] *	CGC
IZ629	* ufsl34[P unc-47 p::mCherry] *	Dr. Michael M. Francis (University of Massachusetts, Worcester, MA)
XQ574	*har-1 ( gk3124 ); unc-47 p::mCherry *	Parker Lab
XQ602	*har-1 ( gk3124 )* ; [myo-3p::TOM20::mRFP + unc-119 (+) + pBS Sk+]	Parker Lab
XQ645	*har-1 ( ad2155* ); [myo-3p::TOM20::mRFP + *unc-119 (+)* + pBS Sk+]	Parker Lab
XQ658	*har-1 ( ad2155 );* *unc-47 p::mCherry *	Parker Lab
